# mTOR Pathways in Cancer and Autophagy

**DOI:** 10.3390/cancers10010018

**Published:** 2018-01-12

**Authors:** Mathieu Paquette, Leeanna El-Houjeiri, Arnim Pause

**Affiliations:** 1 Goodman Cancer Research Center, McGill University, Montréal, QC H3A 1A3, Canada; mathieu.paquette2@mail.mcgill.ca (M.P.); leeanna.el-houjeiri@mail.mcgill.ca (L.E.-H.); 2 Department of Biochemistry, McGill University, Montréal, QC H3G 1Y6, Canada

**Keywords:** mTOR, autophagy, signaling, cancer

## Abstract

TOR (target of rapamycin), an evolutionarily-conserved serine/threonine kinase, acts as a central regulator of cell growth, proliferation and survival in response to nutritional status, growth factor, and stress signals. It plays a crucial role in coordinating the balance between cell growth and cell death, depending on cellular conditions and needs. As such, TOR has been identified as a key modulator of autophagy for more than a decade, and several deregulations of this pathway have been implicated in a variety of pathological disorders, including cancer. At the molecular level, autophagy regulates several survival or death signaling pathways that may decide the fate of cancer cells; however, the relationship between autophagy pathways and cancer are still nascent. In this review, we discuss the recent cellular signaling pathways regulated by TOR, their interconnections to autophagy, and the clinical implications of TOR inhibitors in cancer.

## 1. Introduction

The mechanistic target of rapamycin (mTOR) is a serine-threonine protein kinase that can be divided into two functionally and biochemically distinct complexes, mTORC1 and mTORC2. Both are implicated in growth factor sensing, but mTORC1 is generally the one associated with cell proliferation and cancer progression when deregulated [1]. Significant progress was made in recent years to understand mTORC1 response to growth factors, such as insulin and insulin-like growth factor. The first part of this review highlights important components and pathways upstream of mTORC1. In the following sections, we discuss the mechanisms downstream of mTORC1, specifically those of which mTORC1 promotes growth by negatively regulating autophagy machinery, and link it to several cellular responses.

## 2. Upstream Regulators of mTOR

### 2.1. mTOR Responds to Growth Factors

Growth factors act upstream of mTORC1. Binding of growth factors to their specific receptors triggers a cascade of events leading to increased activity of kinases, such as protein kinase B (PKB or AKT), extracellular signal-regulated kinase (ERK) and ribosomal S6 kinase (RSK), which all ultimately feed into the tuberous sclerosis complex 1 and 2 (TSC1 and TSC2) signaling pathway [2]. These complexes have emerged as critical integrators of growth factors, nutrients and stress signals. Spontaneous and inherited mutations that render TSC1/2 non-functional have been identified as the cause of the tuberous sclerosis complex disorder, which were shown to predispose patients to tumours through hyperactive mTORC1 pathway [3]. TSC1 and TSC2 have distinct activity on mTORC1 signaling and very little sequence homology [4]. However, most studies concentrated their effort on TSC2-dependent mTORC1 regulation. TSC2 integrates signals from various kinases leading to mTORC1 regulation. In particular, AKT phosphorylates TSC2 on five residues (S939, S981, S1130, S1132, and T1462, human proteins) upon growth factor replenishment, which releases TSC2-dependent mTORC1 inhibition [5,6,7,8]. Other kinases can phosphorylate TSC2 with opposite effects. For example, TSC2 phosphorylation by the AMP-activated protein kinase (AMPK) increases its activity, which renders mTORC1 inactive [9,10]. However, TSC2 activity is not directed toward mTORC1, but toward the GTP-binding protein Ras homolog enriched in the brain (Rheb). TSC2 possesses a GTPase-activating proteins (GAP) domain, which stimulates the GTPase activity of Rheb, converting Rheb-GTP to Rheb-GDP for inactivation [11,12,13,14,15,16]. Moreover, TSC2 phosphorylation status does not affect its GAP activity toward Rheb [6,17,18], but rather its spatial localization. Upon growth factor starvation, such as serum withdrawal, TSC2 relocates to the lysosomal membranes in close proximity of mTORC1 [6,19], while growth factor replenishment promotes its dissociation from the lysosomal membranes [5].

Rheb is ubiquitously expressed in all tissues in mammals and anchored at the lysosomal surface, where the GTP-bound form exerts a positive regulation on mTORC1 [13,20,21,22,23]. Structurally, Rheb directly binds to the amino-terminal lobe of the mTORC1 catalytic domain for activation [24]. Moreover, nucleotide-deficient, inactive Rheb mutants trap mTORC1 in a catalytically inactive state [23]. In patients, recurrent mutations in Rheb were identified in publicly-available tumour genome sequencing data of clear cell renal cell carcinoma (ccRCC), the most common form of kidney cancer [25]. In vivo, characterization of these mutations revealed a resistance toward TSC2 GAP activity. In addition, mTORC1 activation is dependent on the presence of another member of the mTORC1 complex, the regulatory associated protein of mTOR (Raptor) [20,26,27,28]. Once activated, the mTORC1 catalytic domain phosphorylates substrates only when bound to Raptor [20,23]. Therefore, the activity of TSC1/2, Rheb, and Raptor are crucial for a correct regulation of mTORC1 in response to growth factors. These multiple inputs for detecting the absence or presence of growth factors depict the fine-tuning required for mTORC1 regulation and cellular adaptation to various situations.

### 2.2. mTOR Senses Amino Acid Levels

The inputs detected by mTORC1 are not limited to growth factors. In addition, information on nutrient abundance, more specifically the amino acids abundance, is relayed to mTORC1. The major proteins regulating mTORC1 activity in regards to amino acid levels are the Ras-related GTPases (Rag) [29]. There are four members (RagA/B/C/D) in mammals, which assemble in heterodimers (A/B with C/D) at the lysosomal surface [30,31]. Their activity is regulated by their guanine nucleotide state; fully-activated Rags harbour GTP-bound RagA/RagB and GDP-bound RagC/RagD [29]. Activated Rags are necessary to anchor mTORC1 at the lysosomes and to allow sensing of amino acids [31]. However, even though Rags were not shown to directly stimulate mTORC1 kinase activity, they seem to modulate mTORC1 localization to other components, such as Rheb, to regulate its activity [32]. Overexpression of constitutively active Rag mutants increased mTORC1 localization to the lysosomes and increased mTORC1 activity even in the absence of amino acids [29,33]. The Rags are tethered to the lysosomal surface to activate mTORC1 by the Ragulator complex, comprising LAMTOR1/p18, LAMTOR2/p14, LAMTOR3/MP1, LAMTOR4/C7orf59, and LAMTOR5/HBXIP [31]. The Ragulator serves as a scaffold [31,34], but has also been shown to act as a guanine-exchange factor (GEF) toward RagA/B, thus promoting Rag activity [34,35]. However, Ragulator requires the presence of the vacuolar H^+^-adenosine triphosphatase ATPase (v-ATPase) complex to detect the abundance of amino acids [34,36,37]. The v-ATPase components assemble and interact with Ragulator only in the absence of amino acids; this complex dissociates when amino acids are replenished [36]. Furthermore, the presence of the v-ATPase is necessary for mTORC1 activation when amino acids are abundant [37]. However, an alternative model has emerged where amino acid transporters, such as the proton-assisted amino acid transporter 1 (PAT1), sense amino acid availability based on their ability to bind to specific amino acids [38,39]. PAT1 exports amino acids from the lysosomal lumen and is an essential mediator of amino acid-dependent mTORC1 activation. PAT1 also physically interacts with the Rag GTPases [38]. In this model, amino acids in the lysosomes are pumped, together with protons, into the cytosol, while the v-ATPase pumps back the protons into the acidic environment of the lysosomes, promoting mTORC1 activation [36,38]. In addition, the v-ATPase serves as an energy sensor [40]. Upon glucose starvation, Liver kinase B1 (LKB1)-AMPK complex is recruited to the v-ATPase-Ragulator complex through the axis inhibition protein (AXIN) [40,41]. AXIN also causes an inhibition of the GEF activity of Ragulator toward the Rags [40].

Leucine and glutamine are specific amino acids sensed to regulate mTORC1 activity. The leucyl-tRNA synthetase (LRS) is the main sensor for intracellular leucine levels. LRS bound to leucine directly binds to RagD, where it acts as a GAP to activate mTORC1 [42,43,44]. Specific mutations in LRS that reduces the binding to leucine block mTORC1 sensing of intracellular amino acid levels [42]. On the other hand, glutamine has been reported to stimulate mTORC1 by a Rag GTPase independent mechanism, in which mTORC1 is recruited to the lysosomes even in RagA/B knockout cells, and required the v-ATPase, but not the Ragulator [45,46]. In addition, glutamine and leucine enhance glutaminolysis and α-ketoglutarate production, which activate mTORC1 [47,48]. These processes are important for cell growth and proliferation, since glutamine and α-ketoglutarate are precursors for other amino acids, nucleotides, and lipids. Conversely, inhibition of glutaminolysis prevented GTP loading of RagB and subsequent activation of mTORC1 [47,48].

Fully-activated Rags and mTORC1 require a GTP-bound RagA/B, which is achieved with Ragulator’s GEF activity, but also require a GDP-bound RagC/D. The tumour suppressor folliculin (FLCN) and its binding partners folliculin-interacting proteins 1 and 2 (FNIP1/2) have been identified as GTPase activating proteins (GAP) of RagD [49,50]. FLCN was shown to bind to the lysosomal surface after amino acid depletion, where it interacted with the Rag GTPases, and was shown to be necessary for mTORC1 activation by amino acids [51]. However, the FLCN-mTOR relationship is unclear because various groups reported mTORC1 activation as well as mTORC1 inhibition in FLCN-deficient cells, suggesting a context-dependent regulation [52,53,54,55,56,57,58,59,60]. More experiments will be needed to clarify the different roles of FLCN in amino acid sensing and mTORC1 regulation. In addition, FLCN plays a role in lysosome positioning. Following serum or amino acid withdrawal, FLCN promotes the peri-nuclear clustering of the lysosomes by promoting the association of Ras-related protein Rab-34 (Rab-34) to the lysosomes [61]. Reciprocally, in nutrient replete conditions and high mTORC1 activity, lysosomes disperse and accumulate at the cell periphery. Interestingly, FLCN heterozygous loss in patients is the cause of the Birt-Hogg-Dubé (BHD) syndrome and predispose to benign cutaneous fibrofolliculomas, bilateral pulmonary cysts, spontaneous pneumothoraxes, and kidney tumours [62]. The exact mechanism leading to cancer progression and the possible role of FLCN-driven mTORC1 regulation still requires further investigation. 

### 2.3. mTOR Responds to Other Stresses

Nutrient availability and energy are also sensed and the information relayed to mTORC1 by another major kinase, the AMP-activated protein kinase (AMPK), via multiple mechanisms. AMPK was shown to phosphorylate TSC2 to inhibit mTORC1 upon specific energy stresses, such as glucose deprivation [9]. However, TSC2-deficient cells remain responsive to AMPK-dependent mTORC1 inhibition by AMPK activators. Therefore, AMPK must regulate mTORC1 by TSC2-independent mechanisms. AMPK was shown to directly phosphorylate the mTORC1 binding partner Regulatory-associated protein of mTOR (Raptor), which leads to its binding to 14-3-3 and mTORC1 inactivation [9]. A third mechanism involves direct phosphorylation and activation of Unc-51 like autophagy activating kinase (ULK1) by AMPK, which in turn binds and inhibits Raptor by phosphorylating it [63]. This third mechanism has been well defined in cellular models, but its importance in mouse models remains to be investigated. The AMPK-dependent regulation of mTORC1 implies that the two protein complexes must be in close proximity. Indeed, a subset of AMPK was shown to reside permanently on the late endosomes/lysosomes [40]. In addition, a recent study suggested that when glucose is absent, the reduction in intracellular fructose 1,6-biphosphate (FBP) and the dissociation of the aldolase from the v-ATPase allow a complex made of AMPK, LKB1, and Axin to bind to the v-ATPase and Ragulator at the lysosomal surface [64]. The now well-positioned activated AMPK at the lysosomal surface can phosphorylate TSC2 and Raptor to antagonize mTORC1 activity.

mTOR was also shown to be regulated by other external stresses, such as oxygen deprivation (hypoxia), radiation, high salt concentration, DNA topoisomerase inhibitors, and histone deacetylase inhibitors [65,66]. Hypoxia is of particular importance in tumourigenesis since early in the tumour environment, an anoxic core builds up in the center of solid tumours. Under hypoxic conditions, the hypoxia-inducible transcription factor (HIF) binds hypoxia-response elements (HREs) to activate the expression of hypoxia-response genes [67]. In contrast, HIF is targeted for degradation in normoxic conditions via the prolyl 4-hydroxylases (PHD) and the von Hippel-Lindau tumour suppressor protein (VHL) [68,69]. Hypoxia-response genes include the regulated in development and DNA damage response 1 (REDD1/DDIT4), which inhibits mTORC1 via TSC1/2. REDD1 releases 14-3-3 dependent inhibition of TSC2 induced by growth factors [70,71,72]. Another hypoxia response gene important for mTORC1 regulation is BCL2/adenovirus E1B 19 kDa protein-interacting protein 3 (BNIP3). BNIP3 was shown to directly bind Rheb and inhibits the mTORC1 pathway [73].

## 3. mTOR Regulates Effectors of the Autophagy-Lysosomal Pathway

The sections above summarize the components and pathways upstream of mTORC1. In the following sections, we discuss the mechanisms downstream of mTORC1 leading to the promotion and inhibition of autophagy.

### 3.1. mTOR Pathways in Autophagy

Macroautophagy (referred to as autophagy hereafter), the cellular self-degradation process, plays an important role in energy supply, particularly during development and in response to nutrient stress. It is a process through which cargo is delivered to double-membrane vesicles, termed autophagosomes, which fuse with the lytic compartment and release the inner vesicle into the lumen, leading to the degradation of cell components and the recycling of cellular building blocks [74,75]. This intracellular mechanism is conserved in eukaryotes from yeast to complex multicellular organisms, and its dysfunction has been implicated in many human diseases, including myopathy, neurodegeneration, and cancer, as well as resistance to pathogen infection [76,77,78,79].

At the molecular level, autophagy plays a context dependent pro-survival or pro-death role by regulating different signaling pathways, including p53, Bax-interacting factor-1 (Bif-1), Beclin 1 (BECN1), ultraviolet irradiation resistance-associated gene (UVRAG), mTOR, protein kinase B (Akt), B-cell lymphoma 2 (Bcl-2), Ras, and Class I PI3K (PI3KI) in cancer [80]. The focus of this part of the review will be mainly on mTOR pathways; however, these pathways are interconnected and they can integrate into an autophagy-related cancer network that could ultimately affect the fate of cancer cells.

Among several components involved in the tight regulation of autophagy, mTORC1, but not mTORC2, has been shown to be a key player in coordinating the respective anabolic and catabolic processes in response to environmental and physiological stresses. Studies have shown that mTORC1 inhibition increases autophagy, whereas stimulation of mTORC1 reduces this process [81]. mTORC2 was reported to indirectly suppress autophagy through the activation of mTORC1. The PI3K signaling axis activates mTORC2, which, in turn, phosphorylates AKT at two different sites, leading to AKT/mTORC1 signaling axis activation [82,83]. Further studies are required to determine whether there is a direct role for mTORC2 in autophagy regulation. In mammals, and under nutrient-rich conditions, it was reported by three independent groups that mTORC1 controls autophagy through the regulation of a protein complex composed of unc-51-like kinase 1 (ULK1), autophagy-related gene 13 (ATG13), and focal adhesion kinase family-interacting protein of 200 kDa (FIP200) through directly phosphorylating and suppressing this kinase complex required to initiate autophagy [84,85,86]. mTORC1 was reported to directly phosphorylate and suppress this kinase complex required to initiate autophagy. Conversely, nutrient withdrawal stimulates the ULK1/ATG13/FIP200 complex formation and initiates autophagy via ULK1 auto-phosphorylation and phosphorylation of its binding partners [84,85,86]. In line with these findings, rapamycin-induced inhibition of mTORC1 was shown to enhance the kinase activity of ULK1, while mTORC1 activation through Rheb overexpression potently represses ULK1 [86]. Subsequent studies further identified Ser758 in the human protein as the major mTORC1-mediated inhibitory phosphorylation site on ULK1, leading to the complex dissociation and autophagy repression [87,88]. In addition to phosphorylation of ULK1, mTORC1 was also shown to indirectly inhibit autophagy through the phosphorylation of autophagy/Beclin-1 regulator 1 (AMBRA1), preventing ubiquitination of ULK1 by TNF receptor-associated factor 6, an E3 ubiquitin protein ligase (TRAF6), which, under starvation conditions, causes ULK1 self-association, stabilization, and enhancement of its kinase activity [89]. 

Another important player in the regulation of ULK1 and mTORC1 autophagy pathway is 5′-AMP-activated protein kinase (AMPK), which is activated upon energy starvation. During conditions of glucose starvation, the ratio of AMP to ATP in eukaryotic cells increases, leading to the activation of AMPK, which in turn binds to and activates ULK1 through direct phosphorylation at Ser317, Ser777, and Ser555 in murine proteins [87,88,90,91,92]. AMPK can further amplify ULK1 activation through several other direct and indirect mechanisms feeding into mTORC1 signaling pathway inhibition, as discussed in the first part of the review. AMPK-activated ULK1, in turn, phosphorylates and inhibits Raptor, leading to mTORC1 inactivation [63,93]. Activation of ULK1 initiates autophagy in part through phosphorylation of AMBRA1 and BECN1 [94,95], which activate VPS34, a class III PI3K, whose activity is crucial for autophagosome formation. VPS34 forms multiple complexes involved in cellular vesicle trafficking and autophagy; however, the ATG14-VPS34 complex is specifically involved in autophagy regulation, causing maturation of autophagosomes from the endoplasmic reticulum [96]. ULK1 was also shown to directly phosphorylate ATG14 in an mTOR-dependent manner, regulating ATG14-VPS34 lipid kinase activity to control autophagy level [97]. In contrast, and in nutrient-replete conditions, mTOR phosphorylates ATG14 in the VPS34 complex and inhibits its lipid kinase activity, providing another level of mTORC1-mediated autophagy inhibition [98]. Moreover, mTORC1 repressive phosphorylation of ULK1 disrupts its interaction with AMPK, thus preventing autophagosome formation and autophagy [87]. In line with this, activated ULK1 was shown to directly phosphorylate AMPK and inhibit its activation, thus providing a negative-feedback loop on autophagy induction [99]. 

In addition to the ULK1 pathway, mTORC1 is likely to impact autophagy through several other mechanisms including the regulation of the death-associated protein 1 (DAP1), a suppressor of autophagy [100], and through WD repeat domain phosphoinositide-interacting protein 2 (WIPI2), a mammalian ortholog of Atg18 (a regulator of early autophagosome formation in yeast), which was identified as potential mTOR effector [101]. Furthermore, recent studies suggest that, in addition to bulk autophagy, selective autophagy can be triggered by ULK1-dependent p62 phosphorylation, enabling the degradation of selective substrates [102].

### 3.2. mTOR Negatively Regulates the MiT/TFE Family of Transcription Factors

mTORC1 also controls autophagy indirectly by negatively regulating the transcription of genes required for lysosome biogenesis. In nutrient-rich conditions, mTORC1 directly phosphorylates and inhibits the transcription factor EB (TFEB), a member of the microphthalmia family (MiTF/TFE) of basic helix-loop-helix leucine zipper transcription factors that controls many genes with key roles in lysosomal function [103,104,105,106]. These studies show that inhibition of mTORC1 leads to the dephosphorylation and nuclear translocation of TFEB, where it binds to the promoter regions of several autophagy genes, and to induce autophagosome biogenesis and autophagosome–lysosome fusion [103,104,105,107]. The mechanisms that regulate TFEB activity were also reported to control the activity of other MiT members such as TFE3 [108,109]. In addition to mTORC1, TFEB, and transcription factor E3 (TFE3) are also activated by different players depending on the environmental cues. Interestingly, AMPK was recently shown to promote autophagy through TFEB activation by blocking the activity of mTORC1 [110], and through increasing the levels of the co-activator-associated arginine methyltransferase (CARM1), an important cofactor for TFEB transcription [111]. Furthermore, the Rag GTPases were shown to bind and sequester TFEB at the lysosomal surface, inhibiting TFEB activity [109]. Conversely, in RagA and RagB deficient cells, TFEB was shown to be constitutively activated regardless of nutrient availability [112]. 

Recent studies have shown that the kinases PLC-1 and DKF-1, the *Caenorhabditis elegans* orthologs of mammalian phospholipase C (PLC) and protein kinase D1 (PRKD1/PKD), respectively, are required for TFEB/TFE3 activation during bacterial pathogen infection [113] and, hence, establishing their additional involvement in the innate immune response and inflammation [113,114,115], which might impact cancer initiation and progression [116]. Thus, the crosstalk between mTORC1 networks and other mediators suggest that they occupy a central position in the regulation of autophagic pathways in cancer contexts. 

### 3.3. mTOR and Autophagy in Cancer

**Figure 1:**
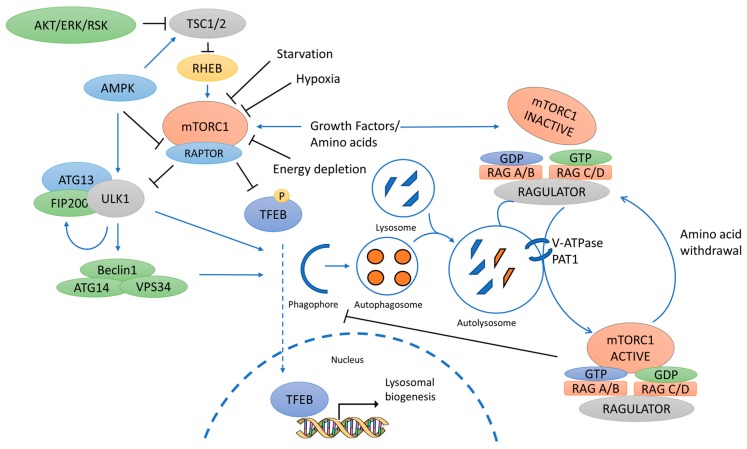
Schematic of key players in the mammalian autophagy pathway discussed in this review. The mechanistic target of rapamycin (mTORC1) is the major control complex for autophagy. A diverse range of signals, such as growth factors and amino acids, regulates mTORC1 by inhibiting the Tuberous sclerosis complex 1 and 2 (TSC1/2), thereby alleviating the inhibitory effect of TSC1/2 on the Ras homolog enriched in the brain (Rheb), which subsequently activates mTORC1. The AMP-activated protein kinase (AMPK) also inhibits mTORC1 via inhibition of the Regulatory associated protein of mTOR (Raptor). mTORC1 is tethered to the lysosomal surface via the Ras-related GTPases (Rags), which activity is regulated by the amino acid sensing of the the vacuolar H^+^-adenosine triphosphatase ATPase (v-ATPase) and the Proton-assisted amino acid transporter 1 (PAT1). Conversion of GTP-GDP is performed by the GTPase-activating proteins/ Guanine-exchange factor (GAP/GEF) activity of Folliculin (FLCN) and Ragulator. Under nutrient-rich conditions, mTORC1 suppresses autophagy by mediating phosphorylation-dependent inhibition of Unc-51 like autophagy activating kinase (ULK1) and the Transcription factor EB/E3 (TFEB/TFE3). Under starvation, ULK1 activation promotes autophagy initiation and autophagosome maturation, and TFEB/TFE3 promote transcription of genes regulating autophagy and lysosomal biogenesis.

The coordinated regulation of autophagy by ULK1, mTORC1, AMPK, and their downstream effectors (Figure 1) may be complex, but it provides a mechanism for autophagy signal integration. Hence, these kinases represent attractive targets for therapeutic treatment. However, the exact role of autophagy in cancer seems ambivalent as both the induction and inhibition of autophagy have been reported to be both pro- and anti-tumourigenic [117]. Specifically, in pre-malignant lesions, considerable evidence suggests that activation of autophagy might prevent cancer development [118]. Conversely, in advanced cancers, both enhancing autophagy and inhibiting it have been suggested as therapeutic strategies [119,120,121]. Autophagy confers stress tolerance, enabling tumour cells to survive under adverse conditions, especially those within the internal mass of tumours, which is usually poorly vascularized [122,123,124,125]. Moreover, autophagy dysfunction is increasingly emerging as a modulator of cancer onset and progression, where it causes the accumulation of damaged macromolecules and organelles, such as the mitochondria and, hence, induces oxidative stress, DNA damage, and chromatin instability [126,127]. Furthermore, stress-induced autophagy in tumour cells was shown to promote drug resistance and tumour dormancy, with eventual tumour regrowth and progression [128]. 

As such, the emergence of autophagy pathways as novel targets for drug development in anticancer therapy have been extensively reviewed [129,130,131,132,133]. Inhibition of autophagy may sensitize the tumour cells to conventional anticancer drugs. However, many cytotoxic and anti-cancer treatments induce autophagy, but none were shown to induce autophagic cell death, per se [134]. Conversely, since cancer cells experience higher metabolic demands and stresses than normal cells [117], they may depend more heavily on autophagy for survival [135] and, thus, induction of autophagy may activate several pathways promoting cell survival, tumour growth, and progression. Accordingly, autophagy induction could be beneficial or detrimental depending on the type or stage of the disease [136] and, subsequently, more studies are required to elucidate the precise function of autophagy in different cancer types before a therapeutic approach can be considered. 

To date, pharmacological induction of autophagy through mTOR inhibition or AMPK activation has been shown to have some therapeutic and prevention potential in cancer [137,138,139]. 

The best-known inhibitor for mTOR is rapamycin. Rapamycin does not directly inhibit the kinase activity of mTORC1 but, instead, it binds, together with a small protein, an immunophilin termed FKBP12, masking the substrate binding site and inhibiting some of its functions [140,141]. The pharmacokinetic properties of rapamycin, itself, were not ideal and, hence, several analogues (known as rapalogs) with improved pharmacological properties were developed to inhibit mTORC1 in anti-cancer therapy for different types of cancer [142]. These rapalogs include temsirolimus (CCI-779), everolimus (RAD001), and ridaforolimus (AP-23573) which are being evaluated in cancer clinical trials for their anti-proliferative functions [143]. However, in terms of autophagy induction, the effectiveness of rapamycin or its analogs were shown to be dependent on cell type, and may sensitize cells to radiotherapy [144,145,146]. However, multiple concerns and limitations emerge from such therapeutic strategies. For instance, using a mouse model of TSC, rapamycin or rapalog treatment to induce autophagy activation was shown to have pro-survival effects for tumourigenesis, suggesting that autophagy inhibition, and not activation, might be effective as a treatment [147]. 

Given the inability of rapamycin and its analogues to inhibit all mTORC1 functions, and its limited efficacy in inducing autophagy as anti-cancer therapy, several compounds inhibiting the kinase activity of mTOR were then developed. These inhibitors were shown to induce autophagy and cytotoxicity in cell and mouse models more efficiently than rapamycin and its analogues [148]. Following their potential success, some of these inhibitors are currently undergoing or completed phase I or II clinical trials as anticancer agents. A new generation of superior mTOR inhibitors containing both rapamycin and an mTOR kinase inhibitor within the same molecule are now being proposed [149]. Pre-clinical studies seem promising in terms of their cytotoxic efficacies compared to rapamycin or mTOR kinase inhibitors alone; however, further studies are necessary to determine whether these beneficial effects can be attributed to increased autophagy. 

Other indirect inhibitors of mTOR (such as metformin) have also been proposed as autophagy-inducers. Metformin activates AMPK indirectly by inhibiting the mitochondrial respiratory chain complex I, thus increasing the cellular AMP/ATP ratio [150]. Therefore, metformin can induce autophagy through the simultaneous activation of AMPK and inhibition of mTOR, and this was shown to have some beneficial effects in various pre-clinical models [151,152,153,154]. 

The therapeutic strategies discussed above are autophagy-inducing through direct or indirect mTOR inhibition. However, the complex/dual role of autophagy in cancer continues to emerge, and more studies are elucidating the beneficial or detrimental outcomes of autophagy-induction depending on the type or the stage of the disease. Hence, further studies are important to elucidate the roles of autophagy in tumour initiation, progression, and metastasis before a therapeutic approach can be considered. 

## 4. Conclusions

As a conclusion, significant progress has been made in the past years in understanding the mTOR signaling pathway and its role in several normal biological processes, as well as in disease. However, much remains to be elucidated in terms of molecular mechanisms that mediate mTOR downstream processes, particularly autophagy. Given the potential double-edged functions of autophagy in tumour suppression and promotion, a better understanding of the different autophagy players and their interplay might provide insights to novel combinatorial therapies aimed at modulating autophagy pathways in cancer to achieve optimal therapeutic benefits.
